# A novel role of LncRNA in regulating tumor metabolism and angiogenesis under hypoxia

**DOI:** 10.1186/s40880-019-0348-x

**Published:** 2019-02-04

**Authors:** Jie Luo, Lee F. Langer, Jian Liu

**Affiliations:** 1 Department of Obstetrics and Gynecology, Women’s Hospital, School of Medicine, Zhejiang University, Hangzhou, 310000 Zhejiang P. R. China; 2 Postdoctoral Research Associate Program, National Institute of General Medical Sciences, National Institutes of Health, Bethesda, MD 20893 USA; 3 Laboratory of Epigenetics and Stem Cell Biology, National Institute of Environmental Health Sciences, National Institutes of Health, Durham, NC 27709 USA; 4 Reproductive and Developmental Biology Laboratory, National Institute of Environmental Health Sciences, Research Triangle Park, Durham, NC 27709 USA

Tumor cells are characterized by metabolic reprogramming, a step that is necessary to support their high demand for energy and nutrients [1, 2]. Furthermore, once metastatic tumor cells are seeded, tumor microenvironment remodeling is immediately initiated, leading to the formation of more suitable conditions for growth. Tumor cells secrete several types of growth factors, cytokines, and chemokines to induce angiogenesis and tumor-associated immune cell recruitment, all of which can promote proliferation and metastasis, as well as trigger immune tolerance and drug resistance [3]. Exploring the synergistic effect between metabolic reprogramming and microenvironment remodeling will likely facilitate the discovery of novel biomarkers and therapeutic targets.

Calcium is an important second messenger in cell signaling modulation. Normally, intracellular calcium performs its signaling functions by activating the calmodulin (CaM) and CaM kinases (CaMKs). The latest research has conclusively demonstrated that calcium flux plays a critical role in tumor progression [4], although how this flux mediates the interconnections between calcium signaling and other pathways is unclear.

Breast cancer is the most common malignancy and the leading cause of cancer death among women worldwide [5]. For this reason, the development of more efficient therapeutic strategies and targets is urgently needed, especially for patients with triple-negative breast cancer (TNBC). A deeper understanding of breast cancer pathogenesis may accelerate the development of therapeutic strategies and targets and thereby improve the outcomes of TNBC patients.

In a recent study published in *Molecular Cell*, entitled “LncRNA *CamK*-*A* regulates Ca(2+)-signaling-mediated tumor microenvironment remodeling” Sang et al. [6], described a novel lncRNA named lncRNA for calcium-dependent kinase activation (*CamK*-*A*), which is shown to mediate the cross-talk between calcium signaling and the nuclear factor kappa-light-chain-enhancer of activated B cells (NF-κB) pathway to promote the Warburg effect, microenvironment remodeling, and tumor development (Fig. 1). The authors identified *CamK*-*A* by RNA interference (RNAi)-coupled glucose uptake and MTT assays using breast cancer cells. By further analyzing the association between *CamK*-*A* expression levels in breast cancer tissues and the survival status of breast cancer patients, *CamK*-*A* expression was found to be significantly associated with cancer progression. Moreover, functional studies, including in vitro viability assays and in vivo xenograft generation assays, showed that *CamK*-*A* promoted tumor growth and enhanced tumor progression.

**Fig. 1:**
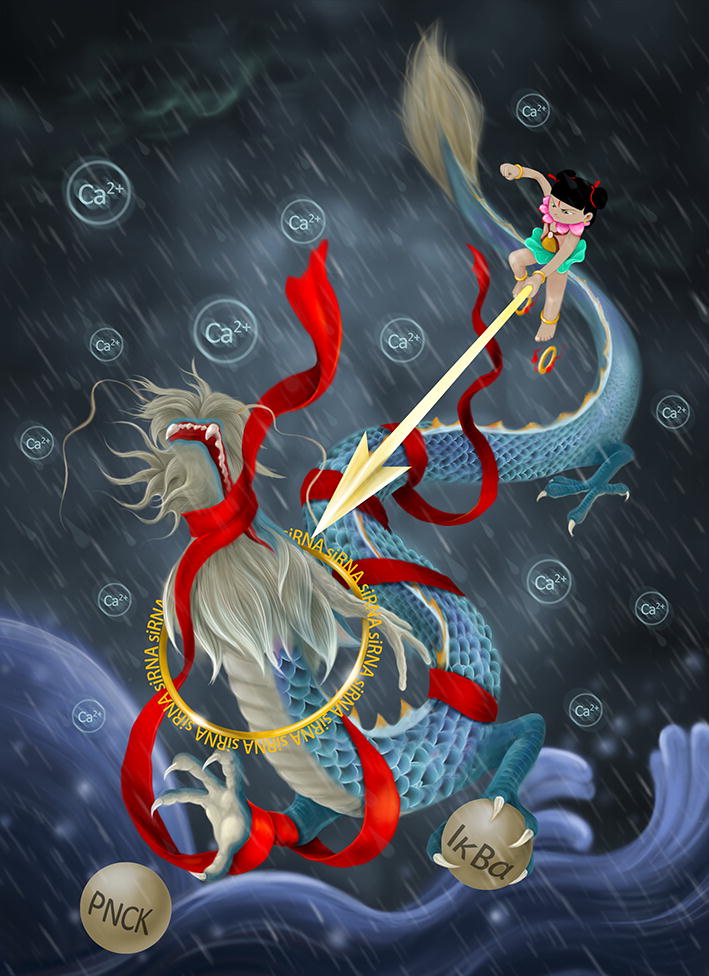
During an unprecedented rainstorm (calcium flux in a hypoxic tumor), the dragon king (lncRNA *CamK*-*A*) holds the two Dragon Balls (PNCK and IκBα), leading to raging seas, representing the tumor microenvironment. Fortunately, the hero Nezha, holding his Hun Tian Ribbon (siRNA) and Qian Kun Collar (RNAi), conquers the dragon king and saves humanity, reflecting the potential application of *CamK*-*A*-targeted therapy in cancer treatment

RNA pull-down followed by mass spectrometry analysis revealed that *CamK*-*A* was a binding partner of pregnancy-up-regulated, non-ubiquitously expressed CaMK (PNCK) and NF-kappa-B inhibitor alpha (IκBα). Using an in vitro kinase assay, the authors demonstrated that *CamK*-*A* facilitated PNCK activation, enabling the kinase to phosphorylate IκBα at Ser32 and ultimately triggering calcium-induced NF-kB signaling activity. In support of this result, a pathway reporter array confirmed that the NF-κB pathway was regulated by *CamK*-*A*. Thus, the authors described a model in which *CamK*-*A* promoted PNCK self-activation, IκBα phosphorylation, and subsequent activation of the NF-κB signaling pathway.

Sang et al. also identified an important link between hypoxia and calcium signaling. Specifically, hypoxia can induce cellular reactive oxygen species production and endoplasmic reticulum stress, leading to increased cytosolic calcium levels. This process is thought to be a crucial microenvironmental element for stimulating solid tumor growth, but the details have remained elusive. Sang et al. demonstrated that by increasing tumor cytosolic calcium concentrations, hypoxia could activate the PNCK-*CamK*-*A*-NF-κB axis and its downstream target genes, including glucose transporter 3 (*GLUT3*), interleukin 6 (*IL*-*6*), interleukin 8 (*IL*-*8*), and vascular endothelial growth factor (*VEGF*). This mechanism induces uncontrolled glucose uptake, tumor-associated macrophage recruitment, intra-tumoral angiogenesis, and tumor microenvironment remodeling.

It is widely assumed that inhibitor of nuclear factor kappa-B kinases (IKKs) phosphorylate IκBα to activate the NF-κB pathway. Therefore, CaMKs are believed to regulate NF-κB through IKKs. However, this study demonstrated that neither knockout nor knockdown of IKK subunit beta (IKKβ) had any effect on PNCK-*CamK*-*A* axis-mediated IκBα phosphorylation. This finding indicates that CaMKs can activate the NF-κB pathway in an IKK-independent manner, providing a major advance in our understanding of calcium-dependent NF-κB pathway activation.

As clinical specimen analysis revealed both that *CamK*-*A* was highly expressed in tumor tissues versus adjacent normal tissues and that its high expression was associated with poor clinical outcomes of patients with breast cancer, Sang et al. examined the role of *CamK*-*A* in tumorigenesis using a patient-derived xenograft model (PDX) of TNBC. In this system, the suppression of *CamK*-*A* using in vivo-optimized RNAi led to robust suppression of tumor proliferation, microvascular tumor growth, macrophage recruitment, and, thereby, tumor microenvironment remodeling. These strong data pave the way for the further clinical application of *CamK*-*A* inhibition. Excitingly, the novel RNAi-based drug, Patisiran (ONPATTOR™) has recently been approved by the US Food and Drug Administration [7], and many more RNAi drugs will likely be developed in the coming years. LncRNA-targeting drugs may contribute to the emergence of RNAi drugs given the tissue specificity of these macromolecules. Exploring *CamK*-*A*’s therapeutic possibilities would be an exciting step along this path.

In summary, the lncRNA *CamK*-*A* activates the calcium signaling pathway under hypoxic tumor conditions, in turn inducing the activation of the CaMK-dependent NF-κB pathway and tumor microenvironment remodeling. Additionally, *CamK*-*A* plays a key role in tumor development, and interference of this lncRNA can robustly block cancer progress, highlighting its potential role in anti-cancer therapy.

## Abbreviations

CaM: calmodulin
CaMKs: calmodulin kinases
TNBC: triple-negative breast cancer
*CamK*-*A*: LncRNA for calcium-depended kinase activation
PNCK: pregnancy up-regulated non-ubiquitously expressed calmodulin kinase
NF-κB: nuclear factor kappa-light-chain-enhancer of activated B cells
IκBα: NF-kappa-B inhibitor alpha
IKKs: inhibitor of nuclear factor kappa-B kinases
GLUT3: glucose transporter 3
IL-6: interleukin 6
IL-8: interleukin 8
VEGF: vascular endothelial growth factor
IKKβ: inhibitor of nuclear factor kappa-B kinase subunit beta
